# Higher brain structural heterogeneity in schizophrenia

**DOI:** 10.3389/fpsyt.2022.1017399

**Published:** 2022-09-23

**Authors:** Keke Fang, Baohong Wen, Lianjie Niu, Bo Wan, Wenzhou Zhang

**Affiliations:** ^1^The Affiliated Cancer Hospital of Zhengzhou University, Henan Cancer Hospital, Zhengzhou, China; ^2^Henan Engineering Research Center for Tumor Precision Medicine and Comprehensive Evaluation, Henan Cancer Hospital, Zhengzhou, China; ^3^Henan Provincial Key Laboratory of Anticancer Drug Research, Henan Cancer Hospital, Zhengzhou, China; ^4^Department of Magnetic Resonance Imaging, The First Affiliated Hospital of Zhengzhou University, Zhengzhou, China; ^5^Department of Breast Disease, Henan Breast Cancer Center, The Affiliated Cancer Hospital of Zhengzhou University, Henan Cancer Hospital, Zhengzhou, China; ^6^Nanyang Institute of Technology, Nanyang, China

**Keywords:** schizophrenia, gray matter volume, heterogeneity, coefficient of variation, personalized

## Abstract

As a highly heterogeneous disorder, schizophrenia shows notable interindividual variation in clinical manifestations. On that account, an increasing number of studies begin to examine the interindividual variability in neuroimaging characterization in schizophrenia. However, whether schizophrenia demonstrates higher interindividual morphological variability than health controls (HCs) remains unknown. T1-weighted anatomical images were obtained from patients with schizophrenia (*n* = 61) and matched HCs (*n* = 73). For each subject, voxel-wise gray matter volume was obtained using voxel-based morphometry analysis. We first inquired whether patients with schizophrenia showed higher interindividual structural variation than HCs using the person based similarity index (PBSI). Then, we examined differences of voxel-wise morphological coefficient of variation (CV) between schizophrenia and HCs. To further associate identified regions showing higher variability in schizophrenia with cognitive/functional processes, functional annotation was performed. Patients with schizophrenia exhibited lower PBSIs than matched HCs, suggesting higher interindividual morphological variability in schizophrenia. The following results showed that patients with schizophrenia exhibited higher CVs than HCs in distributed brain regions including the striatum, hippocampus, thalamus, parahippocampa gyrus, frontal gyrus, and amygdala. Brain regions showing higher CVs in schizophrenia were significantly implicated in affective, incentive and reward related terms. These results provide a new insight into the high clinical heterogeneity and facilitate personalized diagnose and treatment in schizophrenia.

## Introduction

As one of common mentaldisorders, schizophrenia affects more than 20 million people worldwide (1). Although many attempts have been done, the etiology of schizophrenia remains unknown. One of reasons is that schizophrenia is a highly heterogeneous disorder, reflected in diverse clinical presentations (2), complex genetic architectures (3), and various treatment responses (4). The heterogeneity hampers uncovering pathophysiology and discovering validated biomarkers indicative of precision diagnosis and treatment (5) in schizophrenia. Although researchers have consistently found structural aberrance in distributed brain regions with help of modern neuroimaging tools like MRI (6). The interindividual heterogeneity usually leads to discordant findings (7). An increasing number of studies begin to focus on the interindividual heterogeneity in schizophrenia.

The notion that schizophrenia is a heterogeneous disorder is widely accepted and patients with schizophrenia demonstrate considerable interindividual variation in terms of clinical characteristics and neuroimaging abnormalities. For example, although patients with schizophrenia are usually reported to have deficits in intellectual decline after illness onset, there is a fair proportion of patients do not (8). Distinct subtypes of schizophrenia usually demonstrate different or even totally opposite patterns of structural/functional abnormalities (7, 9–11). The heterogeneity is thought to be one of the leading causes that lead to conflicting findings in neuroimaging studies (12, 13). Recently, Brugger and Howes (6) perform a meta-analysis on 108 studies and find that patients with schizophrenia show significantly greater interindividual variation in the volumes of disturbed brain regions including the thalamus, putamen and temporal gyrus than healthy controls. One further research reveals that the high interindividual variation in brain structure is associated with polygenic risk in schizophrenia (14). After these studies, Sun et al. (5) reveal higher interindividual variability of functional connectome in schizophrenia. These results shed lights on the heterogeneity and provide new insights into the high clinical heterogeneity and facilitate personalized clinical decision in schizophrenia. However, whether patients with schizophrenia demonstrate higher interindividual morphological variability than health normal is still unknown.

In this study, we aimed to investigate interindividual morphological variation in schizophrenia. Sixty one patients with schizophrenia and matched healthy controls (HCs, *n* = 73) were included. Voxel-based morphometry analysis (VBM) (15) was performed to obtain voxel-wise gray matter volume for each subject from T1-weighted anatomical image. We first inquired whether schizophrenia showed higher interindividual morphological variation than HCs. Considering the multifarious clinical characteristics and findings of previous studies, we hypothesized that schizophrenia would show higher interindividual morphological variation than matched HCs. Then, we calculated voxel-wise morphological coefficient of variation (CV) measuring the degree of dispersion for each group (16) and obtained voxel-wise differences of interindividual morphological variability between schizophrenia and HCs. Finally, we performed functional annotation to associate identified regions showing higher variability in patients.

## Materials and methods

### Sample

All subjects (both patients with schizophrenia and HCs) come from the Centers of Biomedical Research Excellence available on the Internet^1^ (17). This dataset included 61 patients with schizophrenia and matched 73 HCs. Patients with schizophrenia were diagnosed with the Diagnostic and Statistical Manual of Mental Disorders (DSM-IV, Fourth Edition) for schizophrenia. All patients were on antipsychotic medications and no medication changes in 1 month. None of the patients had a history of neurological disorder, active substance dependence or abuse (except for nicotine) within the past year. Seventy-three healthy controls matched for sex and age were also recruited. All HCs completed a structured clinical interview for DSM-IV axis I disorders-non-patient edition to rule out axis I conditions. The exclusion criteria of HCs included: (1) current or past psychiatric disorder, (2) family history of a psychotic disorder in a first-degree relative, (3) history of more than one lifetime depressive episode, (4) history of depression or antidepressant use within the last 6 months, and (5) history of lifetime antidepressant use for more than 1 year, (6) recent history of substance abuse or dependence, (7) head trauma with a loss of consciousness greater than 5 min. In addition, all subjects refrained from smoking for at least 1 h prior to scanning.

This study was approved by Human Subjects Research Review Committee. Informed consent was obtained from the subjects according to the institutional guidelines at the University of New Mexico.

### Data acquisition

T1-weighted anatomical images of all participants were acquired using Siemens Trio scanner. The main scanning parameters were as followed: echo time = 1.64 ms, repetition time = 2,530 ms, field of view = 256 mm, resolution matrix = 256 × 256, inversion time = 1,200 ms, flip angle = 7 degrees, thickness = 1.0 mm, and voxel size = 1 × 1 × 1 mm^3^.

### Voxel based morphometry analysis

All scans were processed using the CAT12 toolbox.^2^ The standard pipeline steps were adopted mainly including bias-field correction, segmentation gray and white matter and cerebrospinal fluid, adjustment for partial volume effects, normalization into Montreal Neurological Institute space, resampled to 1.5 mm × 1.5 mm × 1.5 mm and non-linear modulation (18–20). Finally, the gray matter (GM) maps were smoothed using 6 mm full width at half maximum Gaussian kernel (FWHM) (21).

### Calculation of the person based similarity index

**FIGURE 1:**
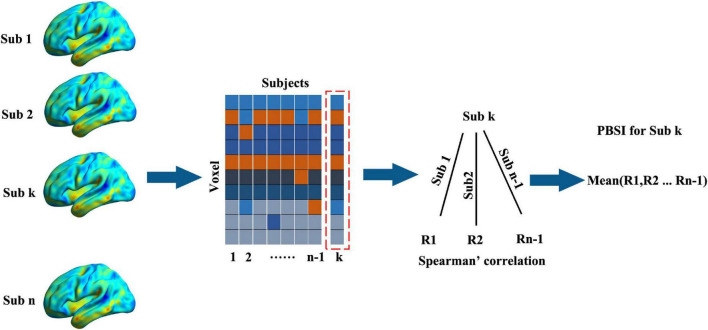
The work flow of calculating the person-based similarity index. The voxel-wise gray matter volumes were concatenated into vectors (voxel × subjects) for one group (the number of subjects was n). For subject k (sub k), the person-based similarity index (PBSI) was the mean Spearman’s correlations (the number was n-1) between it with other subjects.

First, we inquired whether schizophrenia showed higher interindividual morphological variation than HCs by comparing the person based similarity indexes (PBSIs) between them. PBSIs measured the degree of structural similarity between subjects where the higher PBSIs suggested higher similarity in one group (16). There were two main steps to obtain the PBSI. First, the spatial Spearman’s correlations of voxel-wise morphometry between subject k with other subjects in one group were calculated. This step would yield n-1 coefficients for each subject where n is the total number of subjects in the group. Second, averaging the n-1 coefficients to generate the PBSI score for subject k Figure 1. We compared PSBIs between patients with schizophrenia and HCs using two sample *t*-test controlling age and sex.

### Variability of voxel-wise morphometry

The coefficient of variation (CV) for voxel-wise gray matter volume (GMV) was obtained for each group separately according to the following formula: CV = SD/Mean. Mean and SD denoted the mean and the standard deviation of voxel-wise GMVs across subjects in each group. The difference significance was evaluated using the asymptotic test, yielding the statistic value (D) and its *p*-value (22). This procedure was done using cvequality in R-cran. The results were controlled for false positive with *p* < 0.05 (FDR corrected).

### Functional annotation

To associate identified regions showing higher variability in schizophrenia with cognitive/functional processes, functional annotation was performed using brain annotation toolbox (BAT^3^) (23). This procedure used 217 cognitive/functional terms boring clear biological significance (24), that were available on Neurosynth^4^ (25). The significance was determined using permutation test (1000 times) (23) and the permutation *p*-values were corrected for FDR (*p* < 0.05).

## Results

### Demographic information

The demographic information of patients with schizophrenia and HCs was listed in Table 1. More details Information of PANSS was showed in Mayer et al. (17). Patient with schizophrenia exhibited no significant difference with HCs with regards to age and gender.

**TABLE 1:** Demographic information of participants.

	Schizophrenia (*N* = 61)	HCs (*N* = 73)	*P*-value
Age (mean ± SD)	36.57 ± 12.97	36.07 ± 13.08	0.882^a^
Educational level (mean ± SD, years)	4.10 ± 1.49	4.70 ± 1.42	0.116^a^
Gender (female %)	21.31%	31.51%	0.321^b^
Age of onset (mean ± SD, years)	19.73 ± 7.77	-	-
Illness duration (mean ± SD, years)	16.83 ± 13.06	-	-
PANSS positive (mean, SD)	14.60 ± 5.42	-	-
PANSS negative (mean ± SD)	12.63 ± 3.96	-	-
PANSS general (mean ± SD)	27.66 ± 8.74	-	-

^a^Two sample t-test.

^b^Chi-squared test.

PANSS, positive and negative syndrome scale; HCs, healthy controls.

### Lower person based similarity index in schizophrenia

**FIGURE 2:**
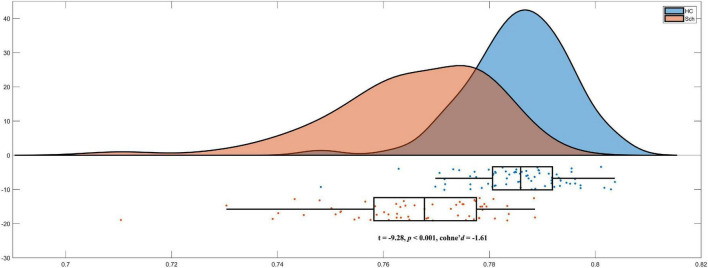
Lower person-based similarity indexes (PBSIs) in schizophrenia compared with healthy controls (HCs).

As we hypothesized, schizophrenia exhibited significant lower PBSIs than HCs (*t* =−9.28, *p* < 0.001, cohen’s *d* =−1.61) Figure 2. These results suggested that patients with schizophrenia exhibited higher morphological heterogeneity than that in matched HCs.

### Voxel-wise morphological variability in schizophrenia

**FIGURE 3:**
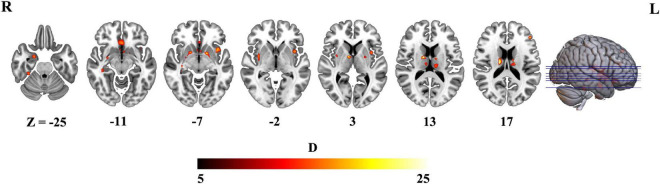
Brain regions exhibiting higher the coefficient of variation (CV) in schizophrenia (*p* < 0.05, FDR corrected).

Then, we further investigated difference between schizophrenia and HCs in variability of voxel-wise morphometry. Patients with schizophrenia exhibited significant higher CVs (*p* < 0.05, FDR corrected) in distributed brain regions including the striatum, hippocampus, thalamus, parahippocampa gyrus, frontal gyrus and amygdala (Figure 3 and Table 2).

**TABLE 2:** Brain regions exhibiting higher coefficient of variation (CV) in schizophrenia (*p* < 0.05, FDR corrected).

Clusters	Peak MNI	Voxels	Including regions	D values
1	–23, -5, –44	163	Left fusiform	19.13
2	33, –35, –26	70	Right fusiform	16.89
3	21, –2, –24	156	Right amygdala	
			Right parahippocampa gyrus	
			Right hippocampus	
4	0, 24, –11	231	Anterior cingulate	16.63
			Medial frontal gyrus	
5	–3, 8, –9	55	Left caudate	14.88
			Anterior cingulate	
6	35, –23, –6	57	Right hippocampus	20.86
7	–15, 3, –8	81	Left putamen	20.10
8	–35, 9, –6	255	Left insula	19.09
9	35, –2, 0	94	Right putamen	18.97
10	14, –3, 12	318	Right thalamus	27.44
			Right caudate	
11	–24, –3, 5	76	Left putamen	15.50
12	24, 3, 12	50	Right putamen	14.68
13	–12, –9, 17	171	Left thalamus	20.24
14	–45, 35, 21	168	Left middle frontal gyrus	25.06
15	–8, 39, 47	58	Left medial frontal gyrus	16.63

D values were the static values of the asymptotic test.

### Functional annotation results

We further performed functional annotation to associate identified brain regions and cognitive/functional processes. Brain regions showing higher CVs in schizophrenia were significantly associated with 14 cognitive/functional processes (permutation *p* < 0.05, FDR corrected), such as affective, incentive and reward related terms (details were included in Table 3).

**TABLE 3:** Functional annotation results.

Functional/Cognitive terms	*P* (FDR corrected)
Affective	<0.001
Anticipation	<0.001
Arousal	<0.001
Incentive	<0.001
Monetary reward	<0.001
Motivation	<0.001
Nociceptive	<0.001
Pain	<0.001
Reactivity	<0.001
Reward	<0.001
Self	<0.001
Self-reported	<0.001
Sleep	<0.001
Stress	<0.001

## Discussion

In this study, we investigated voxel-wise morphological heterogeneity measured with CV in schizophrenia. As we expected, we found significant lower PBSIs in patients with schizophrenia than matched HCs suggesting higher interindividual morphological heterogeneity in schizophrenia. Then, we identified that patients with schizophrenia exhibited higher CVs than HCs in distributed reward-related brain regions. The following functional annotation results suggested that these brain regions were significantly implicated in cognitive/functional processes, such as affective, incentive and reward related terms. These results provided new insights into the heterogeneity of schizophrenia.

The notion that schizophrenia was a highly heterogeneous disorder was increasingly accepted and the heterogeneity was expressed in complex genetic architectures, multifarious phenotypic expressions (3) and variable treatment responses (26). Accordingly, patients with schizophrenia were found to exhibit tremendous interindividual heterogeneity in neuroimaging representations. In a recent meta-analysis, the authors systematically reviewed 108 studies and found that patients with schizophrenia showed higher variability in the volume of distributed brain regions including the amygdala, thalamus and putamen than healthy controls (6). After this study, a series of studies emerged to investigate functional and structural heterogeneity in schizophrenia. For example, Sun et al. (5) identified higher inter-subject variability of the functional connectome in patients with schizophrenia and that the functional variability was associated with clinical heterogeneity. Antoniades et al. (16) found that both patients with schizophrenia and clinical high-risk individuals demonstrated higher brain morphological variation than normal persons. The greater variation aligning with neurobiological heterogeneity was representative of the core feature of the disorder and reflected gene-environment interactions (14). In accordance with these studies, we observed lower PBSIs indicating greater dissimilarity in voxel-wise morphological profiles in schizophrenia. The greater dissimilarity might not be resulted from factors such as medication exposure (16) and suggested that higher interindividual morphological variability was inherent in schizophrenia.

We further identified that brain regions encompassing the striatum, hippocampus, thalamus and parahippocampa gyrus, frontal gyrus, and amygdala exhibited higher CVs in patients than HCs. The higher structural and functional heterogeneity were consistently found in previous studies (5, 6, 14). The higher morphological variation in these regions explained discordant findings in these regions and reflected heterogeneity indicative of putative subtypes. Chand et al. (7) uncovered two subtypes of schizophrenia using a novel semi-supervised machine learning method. These two subtypes exhibited exactly opposite morphological differences in widespread brain regions including brain regions we reported. Consistent with these studies, we observed higher CVs in reward-related brain regions in schizophrenia, reflecting various deficits in reward processing in schizophrenia (27). Notably, Antoniades et al. (16) did not observed significant different in terms of CVs in regional volume between patients with schizophrenia and healthy controls. The reason might be different methodologies used in these two studies. Antoniades et al. (16) measured CVs at regional measures while we focused on voxel-wise CV aberrance. This could be tested in the future. The functional annotation results further revealed that the interindividual morphological variation was related to multifarious reward related deficits in schizophrenia (27).

There were some limitations should be mentioned. First, our results were obtained from one single dataset, future studies could use another independent dataset to confirm our results. Second, we did not have enough clinical data, such as cognitive performance and behavior scores, to establish a relationship between the interindividual variations with clinical characteristics. Third, factors such as cigarette and alcohol were not controlled in this study. Future studies should examine their effects on the morphological variation.

## Conclusion

In this study, we examined the voxel-wise morphological variation in schizophrenia and matched HCs. Patients with schizophrenia exhibited higher interindividual variation than matched HCs. Further analysis showed that schizophrenia demonstrated distributed brain regions had higher morphological CVs than HCs. In addition, the high interindividual variation in these regions was related to multifarious reward related deficits in schizophrenia. Our results provided new insights into the high clinical heterogeneity and facilitated personalized diagnose and treatment in schizophrenia.

## Data availability statement

The original contributions presented in this study are included in the article/supplementary material, further inquiries can be directed to the corresponding author.

## Ethics statement

The studies involving human participants were reviewed and approved by Human Subjects Research Review Committee. The patients/participants provided their written informed consent to participate in this study.

## Author contributions

KF and WZ designed the study. BaW and LN collected the data. KF, BaW, and BoW analyzed the data and drafted the work. LN and WZ revised the draft. All authors contributed to the article and approved the submitted version.

## Footnotes

1
http://fcon_1000.projects.nitrc.org/indi/retro/cobre.html

2
http://dbm.neuro.uni-jena.de/cat12/

3
http://123.56.224.61/softwares

4
https://neurosynth.org/
